# Impact of ionic liquids on absorption behaviour of natural fibers/biopolyethylene biocomposites

**DOI:** 10.1038/s41598-021-99956-9

**Published:** 2021-10-14

**Authors:** Joanna Rakowska, Magdalena Węgrzyn, Ewa Rudnik

**Affiliations:** The Main School of Fire Service, 01-629 Warsaw, Poland

**Keywords:** Environmental sciences, Biomaterials

## Abstract

For many years, there has been a growing interest in technologies enabling the replacement of conventional polymer composites with new materials made from renewable raw materials. It is important to assess the behaviour of biocomposites in various environments, including humid conditions. Recently, ionic liquids have been studied as potential modificators of polymers properties, especially flame retardants. In previous study the impact of ionic liquids on thermal and mechanical properties of biocomposites was assessed. In this study the influence of ionic liquids on moisture absorption properties of biocomposites at different relative humidities (RH) was assessed. The biocomposites were built from polyethylene from renewable resources reinforced with flax or hemp fibers. The effect of the addition of 0.5, 1.0, 2.5 and 5 wt.% phosphonium ionic liquids on the moisture absorption properties of biopolyethylene biocomposite reinforced with natural fibers were tested. Mixtures of biopolyethylene, natural fibers and ionic liquid were calendered at 180 °C and then were compounded by injection moulding. The prepared samples were then characterized for their moisture uptake at 30%, 50% and 100% RH. Moisture absorption by biocomposites depended on the structure of the ionic liquid and the type of fiber. The saturation of moisture of about 0.054% was found for samples modified with tributylethylphosphonium diethyl phosphate and reinforced with flax and hemp fibers at RH 100%. The environmental resistance of the materials was found to be improved after the addition of trihexyltetradecylphosphonium bis (2,4,4-trimethylpentyl) phosphinate. Biocomposites with hemp fibers showed slightly less absorption than with flax fibers. It was also observed that ionic liquids: (bis (2,4,4-trimethylpentyl) phosphinate trihexyltetradecylphosphonate) and (bis (2-ethylhexyl) trihexyltetradecylphosphonium phosphate) protect PE biocomposites with plant fibers against mold in high humidity conditions (RH 100%).

## Introduction

Sustainability is now one of the key drivers of innovation and developing new products. The wide range of applications of polymer products is of great importance for saving petrochemical raw materials. The rate of plastic goods production significantly exceeds the rate of plastic degradation thus causing an imbalance in the environment. Therefore, there is an urgent need to replace conventional petroleum-based plastic with bioplastic to make available simplified dumping and consequently reducing dependency on fossil fuel resources^1–3^. Bio-based polymers utilize plants as an environmentally friendly raw material, which can usually be easily and rapidly degraded^1,4–6^.

To improve the mechanical properties of polymers, some additives, e.g., fillers, are introduced. Biocomposites are described as materials based on polymers matrix derived from natural sources reinforced with natural particles: fibers or grains^4,7–11^. As the matrixes of composites are used biodegradable polymers as polysaccharides, polylactic acid, polyvinyl alcohol^1,7,9^ or not biodegradable biopolyethylene (bio-PE)^2,9^. Polyethylene (PE) is one of the most important products of ethylene chain polymerization. The bio-PE is exploited due to increasing oil prices and environmental awareness. Due to the rising oil prices and ecological awareness, methods for producing bio-PE have been developed. Biobased PE has the same chemical, physical, and mechanical properties as fossil-based PE. The leader in green PE production is Braskem (Brazil, Santelisa Vale), which produces it from sugar cane^12^.

Cellulosic fibers such as flax, hemp, jute, pineapple leaf fiber, banana, sisal are the most common reinforcements used^7,8,13^. These biobased composite materials exhibit enhanced mechanical properties^11^ and additionally, due to the partial replacement of the polymer matrix with cheaper reinforcing materials, are economically attractive^7^.

Fiber-reinforced polymers are very often exploited in products that must be both strong and light enough to meet high demands and increase the sustainability of the process^1,8^. Natural fibers also meet these requirements and have additional advantages. Plant fibers are very desirable in terms of their use in green housing because of their health benefit. According to^14^ hemp fibers can regulate the humidity inside buildings by absorbing and/or releasing water depending on air conditions. Moreover, they are important because of lower pollutant emissions and lower greenhouse gas emissions^3,11,15^ and decreasing environmental impact due to low use of fertilizers and no pesticides^16^.

In response to growing environmental and economic pressures, engineers, architects, constructors are looking for efficient, innovative solutions that save non-renewable raw sources^8^. Various studies have been conducted on the introduction of fillers to improve the mechanical properties of polymers. However, biocomposites design requires matching both biobased matrix and natural fillers. Therefore, incorporation of natural fiber into the biopolyethylene matrix is sustainable due to saving petrochemical raw materials and a promising method to improve the biodegradability of the composite materials^1^.

The hydrophilic nature of fibers, which has a significant influence on their dimensional and structural properties, is a major problem for use this filler as reinforcement in polymers^4,8,10,17^. The hydrophilic nature of the fibers depends on their composition and specific structure, is one of the main factors of the currently limited use and, consequently, the large-scale production of composite from natural fibers, although flax fiber composite sailing yachts or surfboards have been produced^18^. Previous studies of biocomposites reinforced with natural fibers show that the enhancement of mechanical properties is achieved with a filling of up to about 30%^19,20^ or even 40% but as the amount of filling increases, the water absorption of the biocomposites also increases^21,22^. In biocomposites with natural fibers when the fibers are in contact with each other, the main mechanism of moisture absorption is the diffusion process. Whereas at low fiber contents, the primary mechanism is penetration, which depends on discontinuities of the composite structure^22^. Many studies of polymers and polymer composites were carried out using ionic liquids as property modifying substances (ILs)^23–35^. Ionic liquids are defined as chemical compounds with an ionic structure that have a melting point below 100 °C. This property is most often due to the significant difference in size between an organic cation with an extensive, asymmetric structure and a small organic or inorganic anion. This hinders the formation of a homogeneous crystal lattice, and thus significantly lowers the solidification point of the compound^29,36^. Additions of ionic liquids affect the rheological, thermal^32,34^, and flammable properties^33^ of polymers. The use of ILs in polymer science is not limited to their application as solvents. Ionic liquids are also used as additives, including plasticizers, components of polymer electrolytes, and porogenic agents to polymers. Currently, in polymer science, ionic liquids are used not only as solvents but also as components of the polymer matrixes, plasticizers, porogenic agents, components of polymer electrolytes for electrochemical polymerization^28,37^⁠.

The aim of this study is therefore to fill the research gap concerning the influence of ionic liquids modifying the biopolyethylene matrix on the moisture absorption by biocomposites containing 10, 20 and 30% by mass environmentally friendly reinforcement fillers (flax and hemp fibers). Earlier studies showed the possibility of increasing the mechanical and thermal properties of biopolyethylene thanks to the addition of ionic liquids^31^. Therefore, innovative research was undertaken in which composites with flax or hemp as reinforcement and ionic liquids as modifiers of the biopolyethylene matrix were tested under various relative humidity conditions. The tests were carried out in the conditions of relative humidity of 30%, 50% and 100%. For comparison, the influence of phosphonium ionic liquids on the absorption behavior of unfilled biopolyethylene at 50% relative humidity was also investigated.

## Materials

The biopolyethylene HDPE SGF 4960 used in this study was produced by Braskem (Brasil). The minimum biobased content of this grade is 96%, determined according to ASTM D6866 (producers’ information). The flax and hemp fibers (2 mm, 5 mm and 10 mm length) were provided by Ecotex (Poland). The length of the fibers was determined based on research conducted by other authors and technological possibilities^21,30,38,39^. The phosphonium ionic liquids (ILs) used as modifications of properties of bio-composites were prepared by IoLiTec (Ionic Liquids Technologies GmbH Heilbronn, Germany). Data on ionic liquids were shown in Table 1.

**Table 1:** Phosphonium ionic liquids.

Ionic Liquid	Abbreviation	Empirical formula	Molecular weight
Tributylethylphosphonium diethyl phosphate	C1	C_18_H_42_O_4_P_2_	384.47
Trihexyltetradecylphosphonium bis(2,4,4-trimethylpentyl) phosphinate	C2	C_48_H_102_O_2_P_2_	773.27
Trihexyltetradecylphosphonium bis(2-ethylhexyl) phosphate	C3	C_48_H_102_O_4_P_2_	805.2

### Preparation of samples

The bio-composites were compounded in a two-step procedure. Mixtures of bio-polyethylene, natural fibers and ionic liquid were calendaring at 180 °C using Jahnel rolling mill and cut by Retsch cutting mill. The amount of ionic liquid, was 0.5–5 wt.%, while flax and hemp fibers were used in the amount of 10 wt.%, 20 wt.% and 30 wt.% Then bio-composite samples were compounded by injection molding using Arburg 420 M type Allrounder 1000–250 at temperature range: 180–200 °C. The mold temperature was 35 °C.

For comparison, biopolyethylene samples without natural fibers were also prepared.

Biopolyethylene samples modified with ionic liquids were obtained by compounding extrusion using Berstorff two-screw extruder ZE-25-33D (temperature zones: 180–225 °C; screw rotation: 80 rpm). The amount of ionic liquid was 0.5 wt.%, 1.0 wt.%, 2.5 wt.% and 5.0 wt.%.

Compositions of the samples used in this study are summarized in Table 2.

**Table 2:** Composition of the samples.

Sample no	Fiber	Fiber length, mm	Amount of fiber (wt.%)	Ionic liquid	Amount of ionic liquid (wt.%)	Abbreviation
0	–	–	–	–	–	PE
1	–	–	–	C1	1	PE/C1-1
2	–	–	–	C2	1	PE/C2-1
3	–	–	–	C2	5	PE/C2-5
4	–	–	–	C1	5	PE/C1-5
5	–	–	–	C2	0.5	PE/C2-0.5
6	–	–	–	C3	0.5	PE/C3-0.5
7	–	–	–	C3	1	PE/C3-1
8	–	–	–	C3	5	PE/C3-5
9	Flax	2	20	C1	1	PE/FF2-20/C1-1
10	Flax	2	20	C3	1	PE/FF2-20/C3-1
11	Hemp	2	10	C1	1	PE/HF2-10/C1-1
12	Hemp	2	10	C2	1	PE/HF2-10/C2-1
13	Hemp	2	10	C3	1	PE/HF2-10/C3-1
14	Flax	2	20	C2	2.5	PE/FF2-20/C2-2.5
15	Hemp	2	20	C2	2.5	PE/HF2-20/C2-2.5
16	Hemp	2	20	C2	5	PE/HF2-20/C2-5
17	Flax	5	10	–	–	PE/FF5-10
18	Flax	5	20	–	–	PE/FF5-20
19	Flax	5	30	–	–	PE/FF5-30
20	Flax	2	10	–	–	PE/FF2-10
21	Flax	2	20	–	–	PE/FF2-20
22	Flax	2	30	–	–	PE/FF2-30
23	Hemp	2	10	–	–	PE/HF2-10
24	Hemp	2	20	–	–	PE/HF2-20
25	Hemp	2	30	–	–	PE/HF2-30
26	Hemp	10	10	–	–	PE/HF10-10
27	Flax	10	10	–	–	PE/FF10-10
28	Hemp	5	10	–	–	PE/HF5-10
29	Hemp	5	20	–	–	PE/HF5-20
30	Hemp	5	30	–	–	PE/HF5-30

## Methods

### Sorption studies

Moisture sorption measurements of bio-PE-based composites were carried out in different relative humidities (RH) conditions. Before exposure to different moisture, samples were conditioned to 35% RH at 23 ± 1 °C. Pieces of bio-composite materials were placed in desiccators contained demineralized water or saturated salt solutions and maintained at 23 ± 1 °C. The salts used with their corresponding relative humidities were MgCl_2_ (RH 30%), Ca(NO_3_)_2_ (RH 50%) as well as demineralized water (RH 100%)^40^. The specimens for moisture absorption experiments were 10 × 10 × 2 mm. Sorption measurements were carried out using an electronic moisture analyzer MA 60.3Y (Radwag) with a precision of 0.1 mg. Samples were taken once every 2 weeks to check moisture absorption depending on the time until the weight became constant. The drying process enables to a determination moisture content of a given sample by evaporating its free water and other components. The sample's moisture content MA(t) is determined through precise weighing carried out before and after exposure time, calculated using the following equation:

1 $$MA\left(t\right)=\frac{M\left(t\right)-{M}_{o}}{{M}_{o}}*100\mathrm{\%}$$

where MA(t) is the moisture absorption (%) at time *t*, *M*_*o*_ is the oven-dried weight and *M(t)* is the weight of specimen at a given exposure time *t.*

All data from three repeated tests were averaged. The standard deviation values are presented in Table S1 in the Supplementary Information.

## Results and discussion

Thermoplastic polymers, which include polyethylene, are mostly non-polar, which makes them resistant to absorption of moisture^41,42^⁠⁠⁠. Polymer composites with natural fibers are the subject of research on an increasing application due to the advantages of natural fibers. The main application of polymer composites reinforced with natural fibers are construction materials. Research on the moisture absorption behaviour of the bio-composites is very important due to the poor water resistance of fibrous biomaterial. In a different application of bio-composites, mainly for outdoor use, the resistance to water or moisture sorption is one of the key parameters affecting their mechanical properties and dimensional stability^31,43^⁠.

### Influence of kind and size of plant fibers on absorption behaviour of bio-composites

At the first stage, the moisture sorption of bio-composites filled with flax fibers or hemp fibers added in various concentrations (10%, 20%, 30%) with various fiber diameters (2 mm, 5 mm and 10 mm) was studied at 50% relative humidity.

Water sorption studies in polymer composites with natural fibers^8,44^ confirmed that the behaviour of the materials depends on the specific type, size and content of fibers as well as the quality of the interface between the fiber and the matrix, which results from the chemical composition of the fibers, their physical structure and the method of producing the composite determining the distribution of the filling in the matrix. In composite materials, the mechanisms of moisture absorption can be diffusion through the matrix, movement through the pores in the matrix and at the fiber/matrix interface, or capillarity through the fibers^7^.

**Figure 1:**
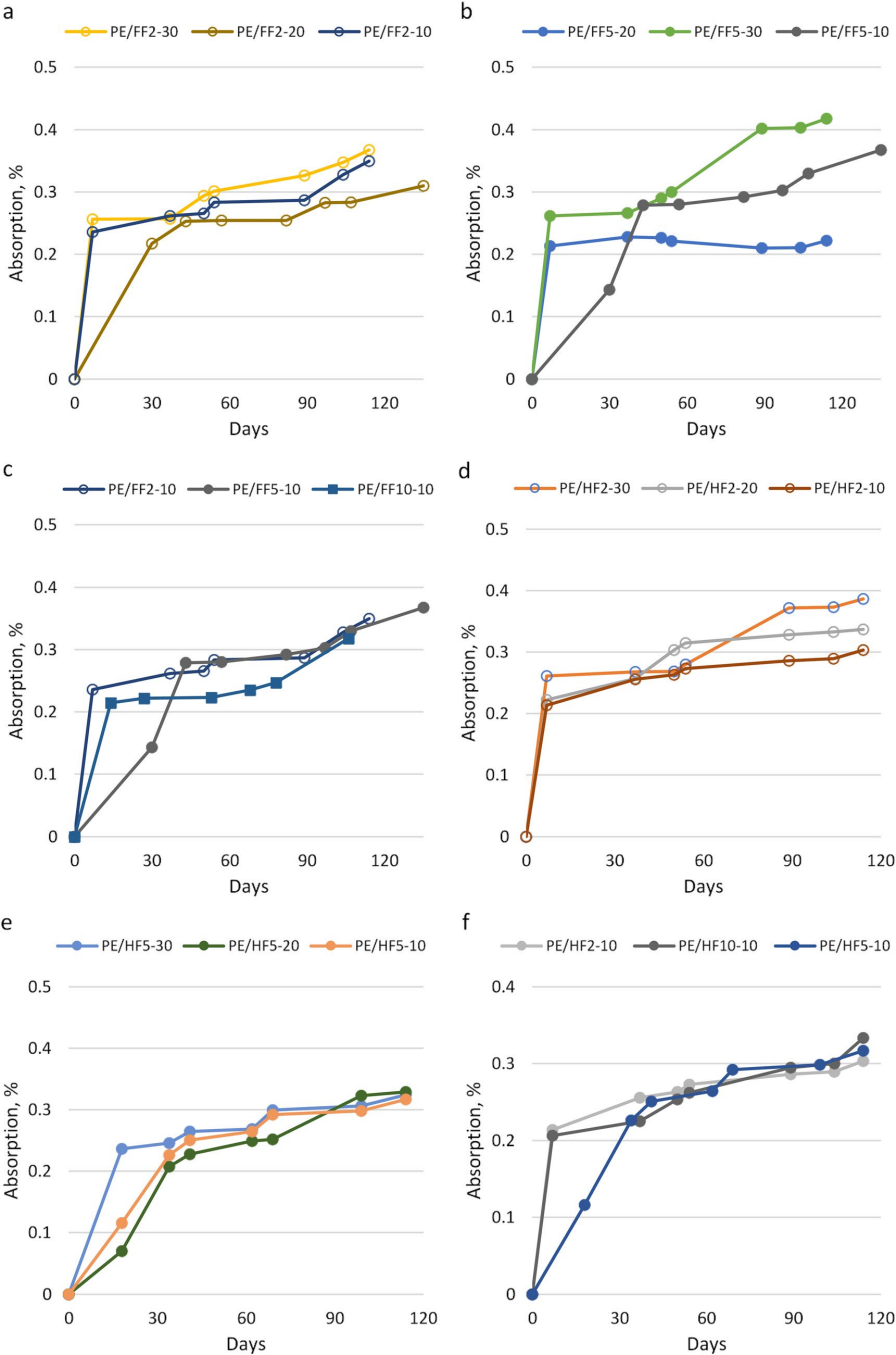
Moisture sorption of bio-composites with flax and hemp fibers at 50% RH; (**a**) flax fiber 2 mm, (**b**) flax fiber 5 mm, (**c**) 10% flax fiber 2 mm, 5 mm, 10 mm, (**d**) hemp fiber 2 mm, (**e**) hemp fiber 5 mm, (**f**) 10% hemp fiber 2 mm, 5 mm,10 mm.

It has been observed that the fiber-based composites showed slightly higher moisture absorption than bio-PE (Fig. 1) due to the hydrophilic nature of flax and hemp with polar hydroxyl and carboxyl groups on the fiber surfaces. These results are in agreement with data published by Parkih^45^ and Sakar^46^ showing that the lignocellulosic fibers displayed a higher tendency to absorb moisture than does the hydrophobic bio-PE. The research of moisture absorption by bio-PE samples reinforced with natural flax and hemp fibers revealed that moisture absorption depended mainly on the amount of added plant fibers when they are in contact with each other, while the length of the tested fibers had a smaller impact on the amount of absorbed moisture.

### Influence of ionic liquids on absorption behaviour of bio-polyethylene matrix

**Figure 2:**
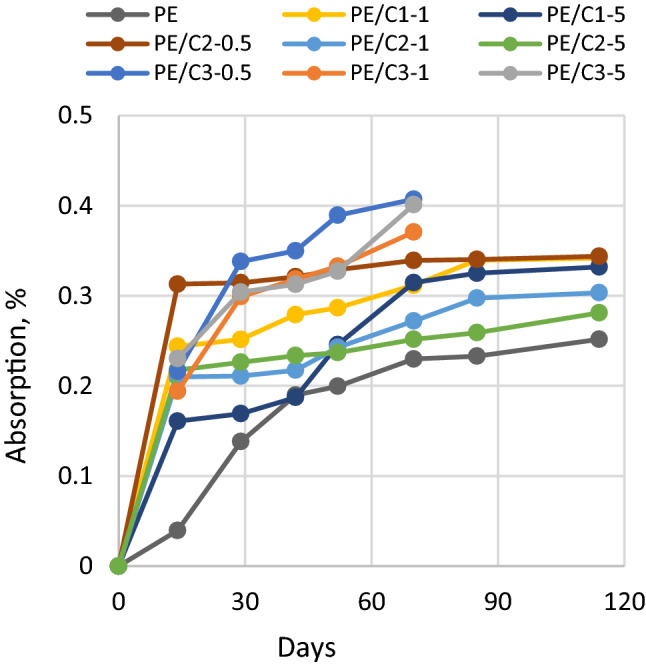
Moisture sorption of bio-PE with ILs at RH 50%.

The influence of ionic liquids on the absorption behaviour of biopolyethylene matrix was studied at 50% RH. Results of moisture sorption by bio-PE matrix modified with various ionic liquids were given in Fig. 2.

The addition of each of the tested ionic liquids increased the sorption of moisture in material samples compared to the pure PE matrix. The smallest increase in moisture absorption was observed for C2-modified biopolyethylene. The highest absorption was observed for C3 modified bio-PE in the whole range of analyzed amounts of ionic liquid, i.e., 0.5%, 1% and 5%. It is noteworthy that for all ionic liquids modified polyethylene samples sorption begins with a rapid initial mass gain, then after 14 days of exposure for C1 and C2 ionic liquids modified PE slowly approaches stabilization, whereas for C3 modified polyethylene samples further moisture uptake was observed. In the analyzed ranges of ionic liquids concentration, no pronounced relationship was found between the amount of modifier addition and the increase in moisture absorption.

### Influence of relative humidity on absorption behaviour of biocomposites modified with ionic liquids

Next, samples of biocomposites modified with ionic liquids were exposed to various relative humidities, i.e., 30%, 50% and 100%.

**Figure 3:**
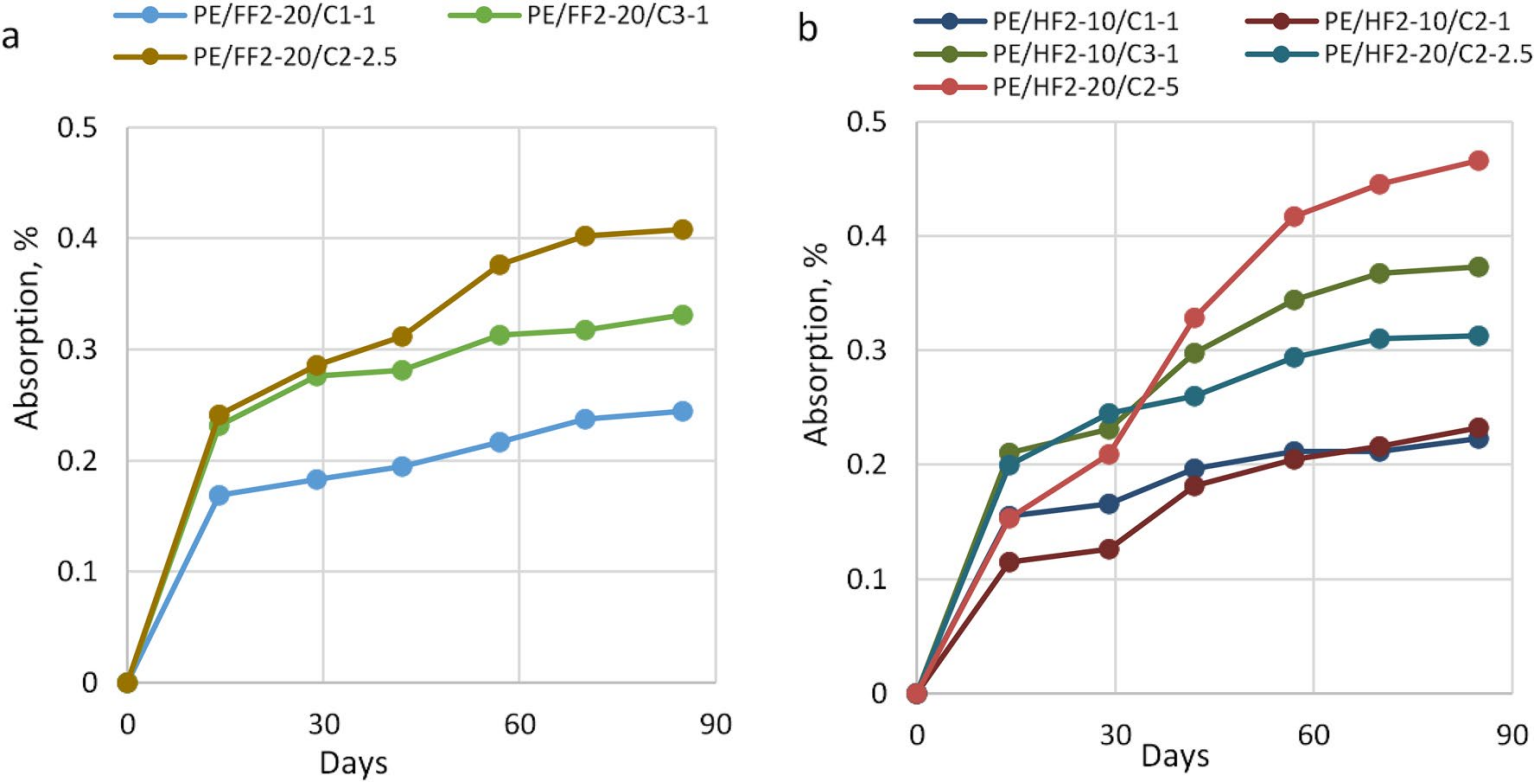
Moisture sorption bio-PE composites with flax or hemp fibers, at 30% RH.

**Figure 4:**
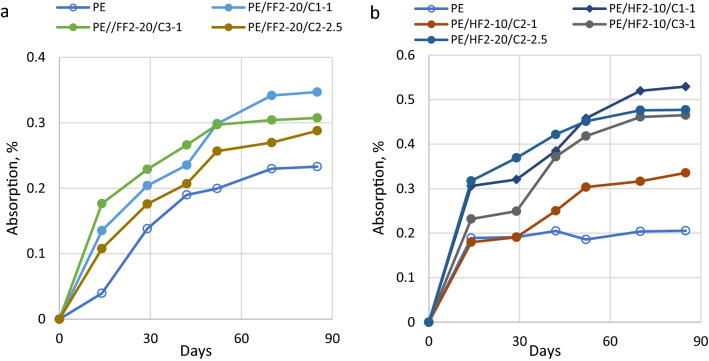
Moisture sorption bio-PE composites with flax or hemp fibers, at 50% RH.

**Figure 5:**
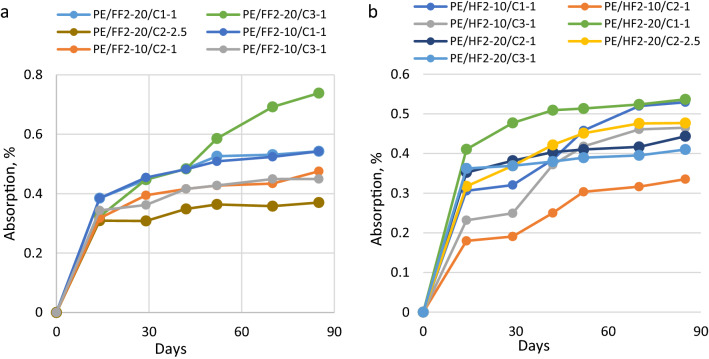
Moisture sorption bio-PE composites with flax or hemp fibers, at 100% RH.

This study focused on the analysis of the moisture sorption properties of the bio-PE composites reinforced with flax or hemp fibers and modified with ILs. The changes of moisture sorption of biocomposites as a function of flax and hemp fiber content and the ILs concentration have been shown in Figs. 3, 4 and 5, respectively at 30%, 50% and 100% relative humidity. It has been found that moisture absorption in all considered material samples increased with increasing humidity levels in the range from 30 to 100%.

At a relative humidity of 30% for the 20% flax reinforced biocomposites the lowest moisture absorption equal 0.244% was observed for biocomposites containing C1 ionic liquid, whereas, for biocomposites reinforced with hemp at 10% level, samples modified with C1 and C2 ionic liquids exhibit the lowest moisture absorption, i.e., 0.223 wt.% and 0.233 wt.%, respectively. Comparing the influence of the kind of natural fiber with the same level of reinforcement (20 wt.%) and the same amount of ionic liquid the higher moisture was observed for flax than for hemp reinforced biocomposites. Composites with a similar amount of ILs based on hemp fibers acquired minor values of water content (0.223–0.373 wt.%) while in flax plant composites slightly bigger water absorbability values were observed (0.244–0.408 wt.%). In the case of biocomposites with hemp fiber, it was observed that the sorption of moisture increased with the increasing addition of IL-C2. The highest moisture content at 30% RH (0.466%) was measured for the sample PE/HF2-20/C2-5 with 5% IL-C2. The results are shown in Fig. 3.

Next, samples of biocomposites modified with ionic liquids exposed at 50% relative humidity were studied. As expected, the moisture sorption for plant fiber-reinforced biocomposites increased in comparison with the absorption of pure biopolethylene matrix (see Fig. 4). In an environment at 50% RH or 100% RH moisture absorption of biocomposites with flax and hemp fibers was similar. At a relative humidity of 50% for the 20% flax as well for 10% hemp reinforced biocomposites the lowest absorption was observed for biocomposites modified with C2 ionic liquid at an amount of 2.5% (Fig. 4). It is noteworthy that regardless of kind of fiber, length of fiber or amount of fiber biocomposites modified with ionic liquid C2 at a concentration of 2.5% exhibit the lowest absorption of moisture.

At a relative humidity of 100% for flax-reinforced biocomposites, the addition of C3 ionic liquid in the amount of 1% results in higher moisture absorption compared to biocomposites containing C2 ionic liquid (IL-C2) both in concentration 1 wt.% and 2.5 wt.% Biocomposites with 10 wt.% hemp filling and 1 wt.% IL-C2 at 100% RH showed 0.335% moisture absorption while the sample with 20% hemp fibers and 2.5% IL-C2 had sorption equal to 0.477%. In this case, it could be due to the influence of the ionic liquid on the properties of the fibers and their adhesion to the biocomposite matrix. Thus, it can be assumed that C2 influences the flexibility/softness of the fibers and the possibility of their arrangement in the composite matrix.

The moisture content in hemp-based biocomposites usually increases with the increase in the average filler concentration (Figs. 3b, 4b, 5b). Samples with flax fibers (Fig. 5a), may behave similarly but confirmation of this thesis requires further research.

Compared to all tested biocomposites, bio-PE matrix filled with hemp fiber and ionic liquids showed lower moisture absorption than biocomposite samples with flax reinforcement (Figs. 3, 4, 5). It can probably be attributed to the differences in the chemical and physical structure of plant fibers and the resulting ability to water sorption on their surface or better transport inside cellulosic molecules. Depending on the plant species, place and conditions of cultivation and technological processing during fiber acquisition, on the surfaces of natural fibers are different types and amounts of waxes (fatty alcohols, hydrocarbons, fatty acids and various esters)^47,48^. Also, the presence of ionic liquids can modify the surface of the fibers and affect their properties, including absorption, strength and biodegradation^47,49,50^.

Therefore, in the process of producing a biocomposite with flax fibers, more space may be formed between the matrix and the fiber than in biocomposites reinforced with hemp fibers. Based on the conducted research, it can be assumed that IL-C3 reduces the adhesion between the fibers and the matrix or increases the stiffness of the fibers, which reduces the adhesion of the biocomposite matrix to the filler. The choice of the filler is important in determining the adhesion between the matrix and the filler^16^. The compact and close packing of filler in bio-PE composites reduce the porosity inside the composites, which in turn contributes to the reduction in water absorption^16^.

The properties of flax and hemp fibers are very similar, and their distinction is often questionable. The morphological characteristics are based mainly on the observation of cross-sections and longitudinal sections of the fibers but do not allow for unequivocal identification. Only the examination of deoxyribonucleic acid allows their differentiation^51^.

### Estimation of the diffusion coefficient

The amount of water molecules absorbed by the composites depends on their diffusion in the matrix. Water diffusion was determined using the diffusion coefficient (D) calculated according to the formula (2):

2 $$D= \pi {\left(\frac{a\cdot h}{4{A}_{\infty }}\right)}^{2}$$

where *a* is the slope of the linear portion of the absorption curve, *h* is the thickness of composite specimens and A_∞_ is the percentage of water absorption at saturation time. The diffusion coefficient determines the rate at which runs the transfer of the diffusing substance across the unit area of section at a certain time. Similar calculations of the water diffusion coefficient in biocomposites with natural fibers are presented in the research^19,52,53^. Estimated values are higher when materials are exposed to higher humidity. Both the modification with ionic liquids and the addition of natural fibers increases the diffusion coefficient. Table 3 contains the diffusion coefficient for samples saturated with moisture.

**Table 3:** Estimated diffusion coefficients.

Sample	Diffusion coefficient at 30% RH (m^2^/s)	Diffusion coefficient at 50% RH (m^2^/s)	Diffusion coefficient at 100% RH (m^2^/s)
PE	7.3653 × 10^–14^	1.8190 × 10^–12^	2.1591 × 10^–12^
PE/C1-1	1.2554 × 10^–12^	No saturated	7.5622 × 10^–12^
PE/C2-1	3.0585 × 10^–13^	1.5168 × 10^–12^	2.0581 × 10^–12^
PE/C2-5	7.9499 × 10^–13^	No saturated	1.7741 × 10^–12^
PE/C3-1	No saturated	6.9843 × 10^–13^	No saturated
PE/C3-5	No saturated	2.3203 × 10^–12^	9.6031 × 10^–13^
PE/FF2-20/C1-1	3.9032 × 10^–13^	1.2086 × 10^–12^	No saturated
PE/FF2-20/C3-1	8.4005 × 10^–13^	1.2460 × 10^–12^	No saturated
PE/HF2-10/C1-1	6.2315 × 10^–13^	1.2287 × 10^–12^	No saturated
PE/HF2-10/C2-1	6.2224 × 10^–13^	6.4529 × 10^–13^	No saturated
PE/HF2-10/C3-1	3.6442 × 10^–13^	8.0996 × 10^–13^	1.4876 × 10^–12^
PE/FF2-20/C2-2.5	3.5716 × 10^–13^	8.8827 × 10^–13^	1.7759 × 10^–12^
PE/HF2-20/C2-2.5	5.8087 × 10^–13^	1.0360 × 10^–12^	1.1307 × 10^–12^
PE/HF5-10	No saturated	No saturated	2.2050 × 10^–12^
PE/HF5-20	No saturated	No saturated	1.5636 × 10^–12^
PE/HF5-30	No saturated	No saturated	1.5586 × 10^–12^

### Visual inspection of biocomposites samples

**Figure 6:**
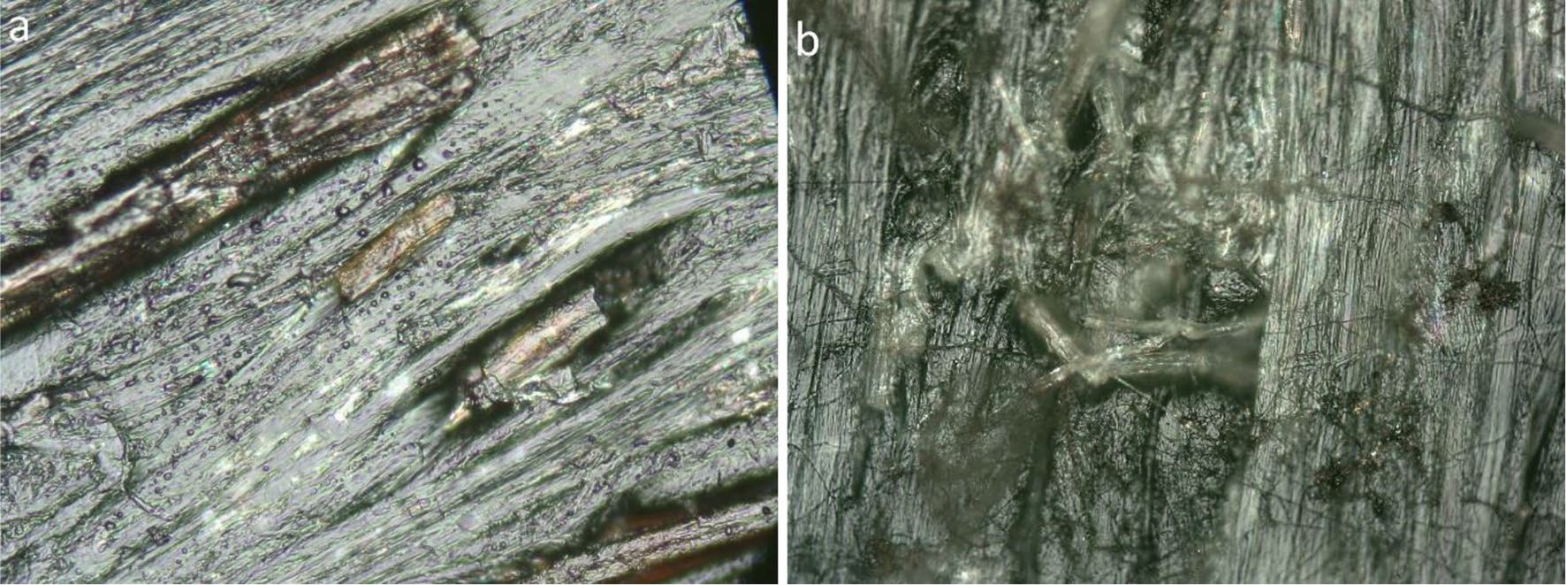
Surface of sample PE/HF2-20/C2-2.5 under magnification 200x; before (**a**) and after saturation (**b**) at 100% RH.

The surfaces morphology of biocomposites was analysed with Optatech MW50 microscope. The samples 10 × 10 × 4 mm were dried in a desiccator for 48 h and then observed with inspected with magnification 50×, 100×, 200× and 500×. The next series of observations were made after the samples had been saturated with moisture. Figure 6 present a surface of sample PE/HF2-20/C2-2.5 with visible fibers extending to the surface of the material, which are channels for moisture transport.

**Figure 7:**
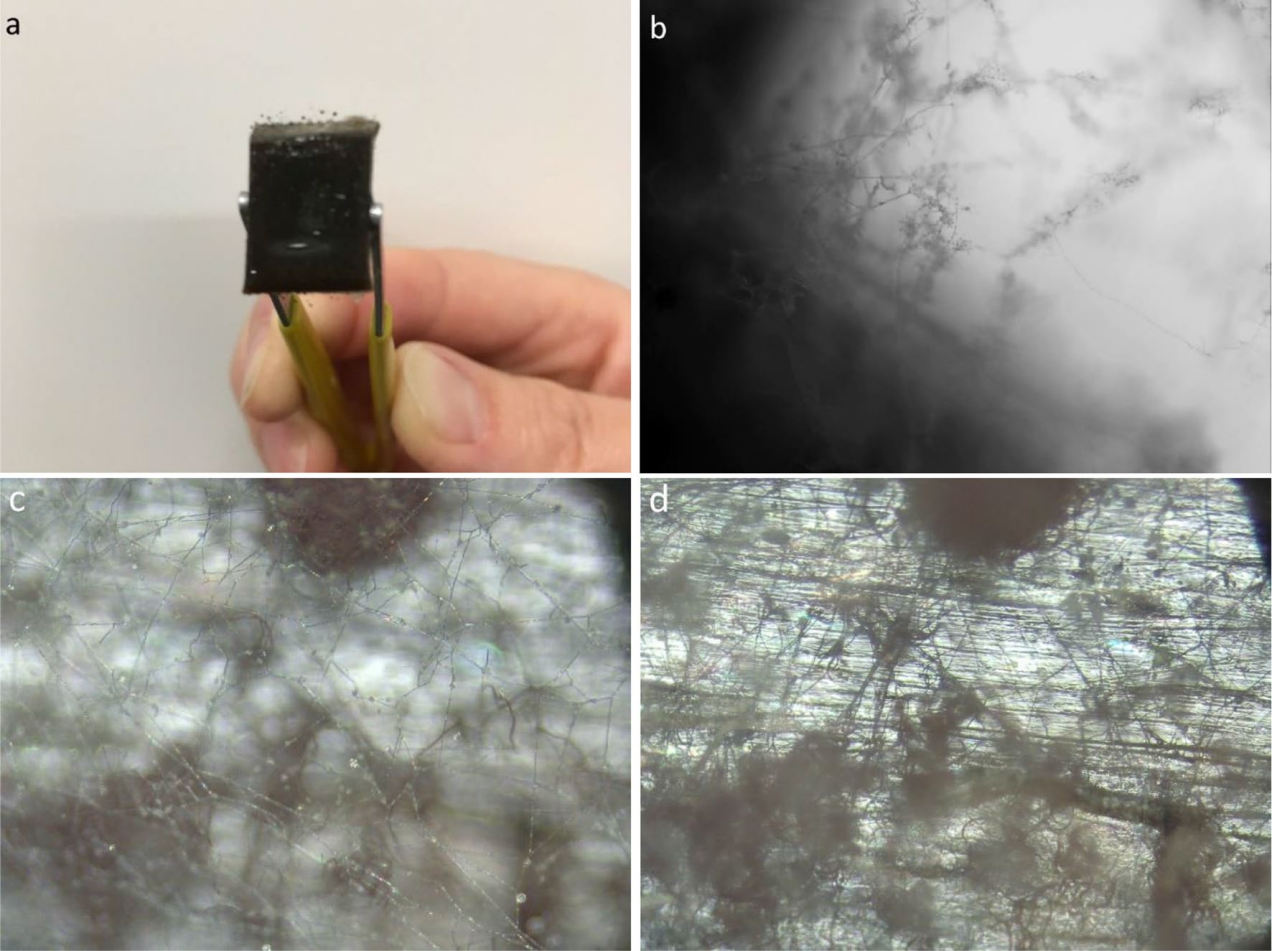
Mold visible on a sample of bio-PE composite with fiber and C1 ionic liquid; (**a**) view of the sample's edge, (**b**) sample's edge with the magnification of 100×, (**c**) a mesh of mold on sample’s surface. magnification 50×, (**d**) surface of the sample under the mold. magnification 50×.

During the periodic measurement of the moisture content in the tested materials after 52 days of exposure to 100% RH humidity on the samples with flax or hemp fibers and IL tributylethylphosphonium diethyl phosphate (C1). The occurrence of mold was observed at the edges. Sample photos are shown in Fig. 7. Based on the observation. It was found that the ionic liquids C2 (trihexyltetradecylphosphonium bis(2,4,4-trimethylpentyl) phosphinate) and C3 (trihexyltetradecylphosphonium bis(2-ethylhexyl) phosphate) probably protect biocomposites filled with plant fibers against mold in high humidity conditions.

To date several publications have shown that ionic liquids may be used as antiseptics. disinfectants and anti-fouling reagents. It was reported that antimicrobial and antifungal activities of ionic liquids are strongly dependent on the length of the substituent alkyl chain while changing the type of anion produced a smaller effect^54^. It may explain the anti-mold behaviour of C2 and C3 modified biocomposites.

## Conclusions

The environmental stability of biocomposite materials containing natural fibers such as flax or hemp is not satisfactory due to their low resistance to moisture absorption. To confirm the suitability of materials for many applications. It is necessary to verify the stability of their properties in various environmental humidity. The sorption behaviour of composites based on flax or hemp and matrix made of bio-PE modified with ionic liquids during storage at room temperature in an environment with different humidity was investigated. This includes examining the effect of flax or hemp fibers. the average length of fibers (2–10 mm) and their amount (10%, 20%, 30%) and the three ionic liquids used at a concentration of 0.5–5%.

The results can be summarized as follows:

1. Research on changes in moisture content in bio-PE composites with flax or hemp fiber filling and an alternative addition of ionic liquids showed better environmental resistance of materials with the addition of trihexyltetradecylphosphonium bis (2,4,4-trimethylpentyl) phosphinate (C2).
2. The addition of ionic liquids increased the absorption of moisture of samples reinforced with flax and hemp fibers, especially in conditions of exposure to very high humidity. Biocomposites with hemp fibers were characterized by a lower moisture absorption than with flax fiber.
3. Based on the conducted research, no pronounced relationship between the fiber length and the biocomposite absorption was found but the moisture absorption depended mainly on the amount of added plant fibers when they are in contact with each other. The fibers sticking out the surface of the composite facilitate the transport of moisture inside the material.
4. It was found that the ionic liquids trihexyltetradecylphosphonium bis(2,4,4-trimethylpentyl) phosphinate (C2) and trihexyltetradecylphosphonium bis(2-ethylhexyl) phosphate (C3) protects PE biocomposites with plant fibers against the occurrence of mold in conditions of high humidity (RH 100%).
5. The adhesion of biocomposites components and its effect on moisture absorption is an important factor that should be taken into account in further research.

## Supplementary Information

Supplementary Table S1.
