# Supplementary material for: Impact of ionic liquids on absorption behaviour of natural fibers/biopolyethylene biocomposites
Source: Sci Rep. 2021 Oct 14;11:20483. doi: 10.1038/s41598-021-99956-9 (PMC8516865; doi:10.1038/s41598-021-99956-9)
Supplement: Supplementary file 1 — Supplementary Table S1. [file 41598_2021_99956_MOESM1_ESM.docx]

**Supplementary information**

Table S1. Standard deviation of absorption

| Sample | Standard deviation of absorption |
| --- | --- |
| PE | 0.0462 |
| PE/C1-1 | 0.1425 |
| PE/C1-5 | 0.0413 |
| PE/C2-0.5 | 0.0933 |
| PE/C2-1 | 0.0275 |
| PE/C2-5 | 0.0852 |
| PE/C3-0.5 | 0.0288 |
| PE/C3-1 | 0.0441 |
| PE/C3-5 | 0.0085 |
| PE/FF2-20/C1-1 | 0.0409 |
| PE/FF2-20/C3-1 | 0.0992 |
| PE/HF2-10/C1-1 | 0.0571 |
| PE/HF2-10/C2-1 | 0.0843 |
| PE/HF2-10/C3-1 | 0.0006 |
| PE/FF2-20/C2-2.5 | 0.0701 |
| PE/HF2-20/C2-2.5 | 0.0035 |
| PE/HF2-20/C2-5 | 0.0317 |
| PE/FF5-10 | 0.0412 |
| PE/FF5-20 | 0.0082 |
| PE/FF5-30 | 0.0165 |
| PE/FF2-10 | 0.0599 |
| PE/FF2-20 | 0.1252 |
| PE/FF2-30 | 0.0219 |
| PE/HF2-10 | 0.0681 |
| PE/HF2-20 | 0.0191 |
| PE/HF2-30 | 0.0728 |
| PE/HF10-10 | 0.0835 |
| PE/FF10-10 | 0.0041 |
| PE/HF5-10 | 0.0366 |
| PE/HF5-20 | 0.0472 |
| PE/HF5-30 | 0.0903 |
